# Safety and efficacy of apixaban versus vitamin K antagonists in patients undergoing dialysis: a systematic review and meta-analysis

**DOI:** 10.1080/0886022X.2024.2349114

**Published:** 2024-05-21

**Authors:** Yifang Zhang, Jialiang Wang, Nannan Shen, Jie Jiang, Yanna Xie

**Affiliations:** Affiliated Hospital of Shaoxing University, Shaoxing City, China

**Keywords:** Hemodialysis, end-stage renal disease, direct oral anticoagulants, warfarin, bleeding

## Abstract

**Background:**

This review aims to evaluate the safety and efficacy of apixaban vs. vitamin K antagonists (VKAs) in patients on dialysis.

**Methods:**

All types of studies published on PubMed, Embase, CENTRAL, and Web of Science up to 10 September 2023 and comparing outcomes of apixaban vs. VKA in dialysis patients were eligible.

**Results:**

Two randomized controlled trials (RCTs) and six retrospective studies were included. Apixaban treatment was associated with significantly lower risk of major bleeding (RR: 0.61; 95% CI: 0.48, 0.77; *I*^2^ = 50%) and clinically relevant non-major bleeding (RR: 0.82, 95% CI: 0.68, 0.98, *I*^2^ = 9%) compared to VKA. Meta-analysis also showed that the risk of gastrointestinal bleeding (RR: 0.74, 95% CI: 0.64, 0.85, *I*^2^ = 16%) and intracranial bleeding (RR: 0.64, 95% CI: 0.49, 0.84, *I*^2^ = 0%) was significantly reduced with apixaban. Meta-analysis showed no difference in the risk of ischemic stroke (RR: 0.40, 95% CI: 0.06, 2.69, *I*^2^ = 0%), mortality (RR: 1.26, 95% CI: 0.74, 2.16, *I*^2^ = 94%) and recurrent venous thromboembolism (RR: 1.02, 95% CI: 0.87, 1.21, *I*^2^ = 0%) between the two groups. Subgroup analysis of RCTs showed no difference in bleeding outcomes.

**Conclusions:**

Low-quality evidence from a mix of RCTs and retrospective studies shows that apixaban may have better safety and equivalent efficacy as compared to VKA in dialysis patients. Apixaban treatment correlated with significantly reduced risk of major bleeding and clinically relevant nonmajor bleeding in observational studies but not in RCTs. The predominance of retrospective data warrants caution in the interpretation of results.

## Introduction

Anticoagulation is frequently required for prophylaxis of thromboembolic episodes in patients with atrial fibrillation (AF) as well as venous thromboembolism (VTE) [1]. Until recently, vitamin K antagonists (VKAs) like warfarin and phenprocoumon were the only anticoagulant drugs available for oral use. These drugs are safe and effective if the correct therapeutic range can be achieved for the maximal time. However, achieving a correct international normalization ratio (INR) is often difficult due to food and drug interactions as well as liver diseases [2]. Direct oral anticoagulants (DOACs), such as dabigatran, rivaroxaban, apixaban, and edoxaban, have recently emerged as an alternative option for patients requiring oral anticoagulation [3]. These drugs have rapid onset of action, fixed dosage, and minimal dietary interactions which make them an attractive option as they do not require constant monitoring [2]. Several studies have shown that DOAC and VKA have comparable safety in several patient subgroups [4–6]. However, there is less clarity on the efficacy of these drugs on special cohorts, such as patients with end-stage renal disease (ESRD) who require anticoagulation for various indications [7]. Patients with ESRD who undergo dialysis have a higher risk of bleeding and thrombosis as compared to patients with normal renal function [8]. Anticoagulation in these patients is often challenging and there is limited evidence on the efficacy and safety of DOAC vs. VKA in this cohort. Prior randomized controlled trials (RCTs) comparing DOAC with VKA have excluded patients on dialysis which limits the generalizability of the findings to these patients [9,10]. Concerns with the use of DOAC in dialysis patients stem from the fact that the drug activity of DOAC can be unpredictable in the case of ESRD. Also, there is no reliable measurement of drug activity for DOAC. Apixaban, one of commonly used DOAC, is eliminated via multiple pathways which include metabolism, biliary excretion, and direct intestinal excretion. Only about 27% of the drug is cleared via renal excretion [11] which is why apixaban has been used for dialysis patients. Previously, a few retrospective studies have compared outcomes of apixaban and VKA but with inconsistent results [12,13]. Moreover, previous systematic reviews and meta-analyses did not focus exclusively on apixaban, and included a limited number of studies which limits the strength of the evidence [14–17]. Murtaza et al. [18] conducted a meta-analysis of four studies comparing apixaban and warfarin in patients on hemodialysis and concluded that apixaban may have better safety profile. Another 2023 review by Shen et al. [7] that included only five studies conducted a network meta-analysis to assess the safety of different DOAC in patients on dialysis and showed that apixaban cannot be recommended for mortality reduction or for preventing ischemic or hemorrhagic strokes. Given the conflicting evidence and low number of studies in previous reviews, there is a need for more up-to-date evidence. In this study, we present the results of the most updated meta-analysis comparing only apixaban with VKA in dialysis patients who require anticoagulation.

## Materials and methods

### Literature search and selection criteria

The protocol was published on PROSPERO (CRD42023455862). An electronic literature search was done on PubMed, Embase, CENTRAL, and Web of Science databases for articles on anticoagulant use in dialysis patients. Two reviewers (YX and YZ) searched the web separately for studies published up to 10 September 2023. The PRISMA guidelines [19] were employed during the review (Supplementary Table 1). Databases were searched using various combinations of ‘direct oral anticoagulants’, ‘DOAC’, ‘novel oral anticoagulants’, ‘NOAC’, ‘apixaban’, ‘warfarin’, ‘Vitamin K antagonist’, ‘phenprocoumon’, ‘hemodialysis’, ‘dialysis’, ‘end-stage renal disease’, and ‘renal failure’. Further details are provided in Supplementary Table 2. YX and YZ concluded the search and organized the data in the reference software. Deduplication was undertaken, and titles and abstracts of all articles were then screened for eligibility.

The inclusion criteria were:

1. Studies conducted on patients undergoing any type of dialysis requiring anticoagulation for any reason.
2. Studies comparing apixaban with VKA.
3. Studies reporting bleeding, thromboembolic and mortality data.
4. All observational studies and RCTs were eligible.

We excluded studies not reporting separate data on apixaban, not exclusively on dialysis patients, and those not reporting any of the required outcomes. Reviews, abstracts, and non-English language studies were also excluded.

Articles were selected for full-text review by the two reviewers based on these criteria, and further discussed with another author (JW) for final inclusion in the review. References of other included articles were also screened to identify additional relevant papers. All disagreement was resolved in discussion with the third author (JW).

### Data extraction and study quality assessment

Extracted data included details of the primary author, year of publication, study location, study type, indication for anticoagulants, type of VKA, dose of apixaban, sample size, male gender, coronary artery disease, diabetes, previous myocardial infarction, CHA2-DS2-VASc risk score, New York Heart Association class III/IV, antiplatelet users, and follow-up.

Outcomes of interest were major bleeding, clinically relevant nonmajor bleeding, gastrointestinal (GI) bleeding, intracranial bleeding, ischemic stroke, mortality, and recurrent VTE. All definitions used by the studies were accepted and none of the outcomes were predefined.

Risk of bias assessment was done using Cochrane risk of bias-2 tool [20] for the RCTs, and the Newcastle Ottawa Scale (NOS) [21] for retrospective studies. Two reviewers (JW, NS) conducted the risk of bias assessment independently of each other. In case of disagreement, a third reviewer was consulted (JJ). We also assessed the certainty of evidence based on the Grading of Recommended Assessment, Development and Evaluation system using the Gradepro online tool (https://www.gradepro.org).

### Statistical analysis

Quantitative synthesis was carried out by ‘Review Manager’ (RevMan, version 5.3; Nordic Cochrane Center (Cochrane Collaboration), Copenhagen, Denmark; 2014). All dichotomous outcomes were pooled to obtain risk ratios (RRs) with 95% confidence intervals (CIs). Forest plots were produced in the software by using the random-effect meta-analysis model. Statistical heterogeneity between studies was examined using the *I*^2^ test. Statistically, significant heterogeneity was defined as an *I*^2^ statistic >40%. All analyses were conducted using a random-effects model. Funnel plots were constructed for publication bias. Sensitivity analysis was done by removing one study at a time in Review Manager. Subgroup analysis was done based on study design. A qualitative synthesis of data was conducted for bleeding outcomes based on apixaban dose.

## Results

### Search results

**Figure 1.**
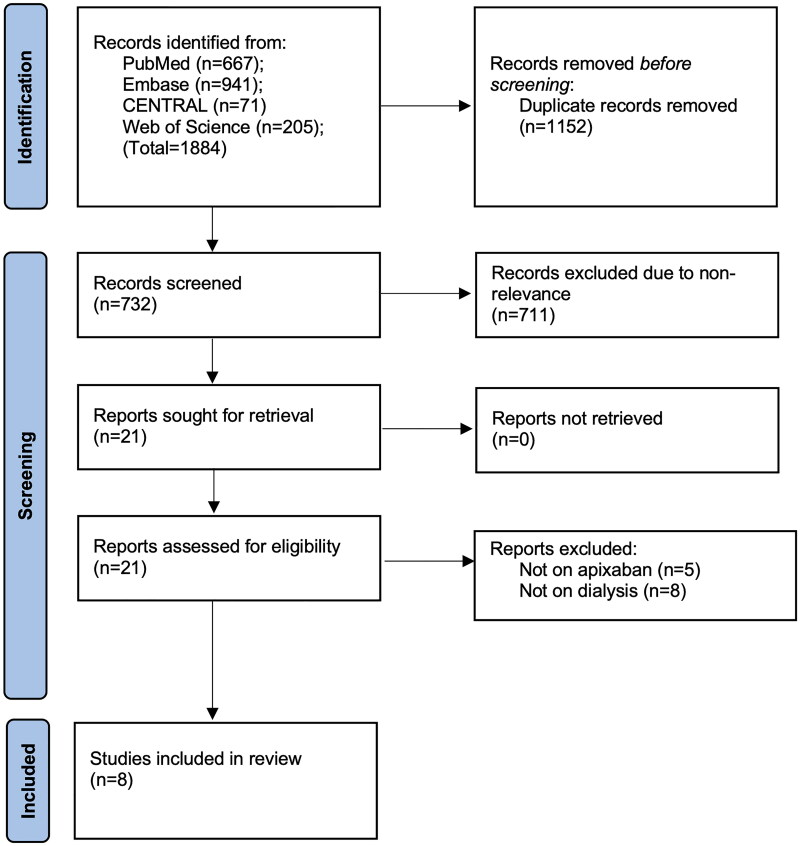
Study flowchart.

Figure 1 demonstrates the search outcomes at every step. Literature search identified a total of 1884 studies. Of them, 732 studies were unique and were examined further by the reviewers. A total of 21 were selected for the full-text examination. Eight [12,13,22–27] met the inclusion criteria and the rest (13 studies) were excluded as they did not exclusively report on apixaban or dialysis patients. Kappa values for inter-reviewer agreement for selection of studies were high (0.9).

### Baseline details

Six retrospective studies and two RCTs were included in the analysis (Table 1). All studies were published between 2017 and 2023. Both RCTs reported data of patients with AF. The retrospective studies included patients with AF or VTE. One RCT used a 2.5 mg dosage of apixaban while the other used 5 mg. Both dosages were used in the retrospective studies in various proportions. All except one study used warfarin in the control group. One RCT compared apixaban with phenprocoumon. Pooled sample size of all studies was 22,405 with a total of 5108 patients treated with apixaban and 17,297 treated with VKA. The use of additional antiplatelet drugs was not significantly different between the two groups. There was a variability in the follow-up duration among the studies. All studies defined outcomes using the standard definition of the International Society of Thrombosis and Haemostasis consensus [28].

**Table 1.** Details of included studies.

Study	Type	Patient population	VKA	Apixaban dose	Groups	Sample size	Mean age (years)	Male gender (%)	CAD (%)	Diabetes (%)	Previous MI (%)	CHA2-DS2-VASc score	NYHA class III/IV (%)	Aspirin users (%)	Clopidogrel users (%)	Follow-up
Reinecke et al. [23]	RCT	AF	PC	2.5 mg BD	Apixaban VKA	48 49	74.7 74.8	64.6 75.5	33.3 23.1	NR	18.8 24.5	5[3.5–5] 4.5[4–6]	33.3 22.5	33.3 34.7	NR	429 days
Pokorney et al. [22]	RCT	AF	W	5 mg BD^a^	Apixaban VKA	82 72	69 68	58.5 69.4	NR	51.2 65.3	19.5 30.6	4[3–5] 4[3–5]	21.4 10.5	36.7 45.7	2.5 1.4	330–340 days
Ellenbogen et al. [25]	R	VTE	W	2.5 mg and 5 mg BD^b^	Apixaban VKA	2302 9263	59.8 58.3	44.7 45.9	55.1 54.2	69.2 66.3	NR	NR	NR	0.6 0.7	NR	6 months
Ionescu et al. [24]	R	AF, VTE	W	2.5 mg and 5 mg BD^c^	Apixaban VKA	144 563	68.8 67.2	56.3 59.1	57.6 65.7	63.9 67.3	NR	NR	NR	50 (all antiplatelets) 50.8		Up to 5 years
Schafer et al. [27]	R	AF, VTE	W	2.5 mg and 5 mg BD	Apixaban VKA	91 103	NR	NR	NR	NR	NR	NR	NR	NR	NR	Up to 1 year
Reed et al. [12]	R	AF, VTE	W	2.5 mg and 5 mg BD^d^	Apixaban VKA	74 50	59.5 62	51.4 62	NR	NR	NR	4.1 ± 1.2 4 ± 1.4	NR	28.4 36	NR	10 months
Siontis et al. [26]	R	AF	W	2.5 mg and 5 mg BD^e^	Apixaban VKA	2351 7053	68.9 68	54.4 54.9	NR	75.4 73.2	NR	5.27 ± 1.77 5.14 ± 1.78	NR	6.6 (all antiplatelets) 7		Up to 5 years
Sarratt et al. [13]	R	VTE	W	2.5 mg and 5 mg BD^f^	Apixaban VKA	40 120	70.9 66.5	50 48.3	50 46.6	45 50.8	NR	4.25 ± 1.43 4.75 ± 1.42	NR	NR	NR	NR

AF: atrial fibrillation; CAD: coronary artery disease; RCT: randomized controlled trial; VKA: vitamin K antagonist; PC: phenprocoumon; W: warfarin; R: retrospective; VTE: venous thromboembolism; NYHA: New York Heart Association.

^a^ 2.5 mg for patients ≥80 years of age, weight ≤60 kg, or both.

^b^ 50.0% used 5 mg, 40.5% used 2.5 mg tablets, and 9.5% used mix of both.

^c^ Fifty-two patients used 5 mg and 92 patients used 2.5 mg.

^d^ 79.7% used 5 mg and 20.3% used 2.5 mg.

^e^ One thousand and thirty-four patients used 5 mg and 1317 patients used 2.5 mg.

^f^ Seventeen patients used 5 mg and 23 patients used 2.5 mg.

### Meta-analysis

**Figure 2.**
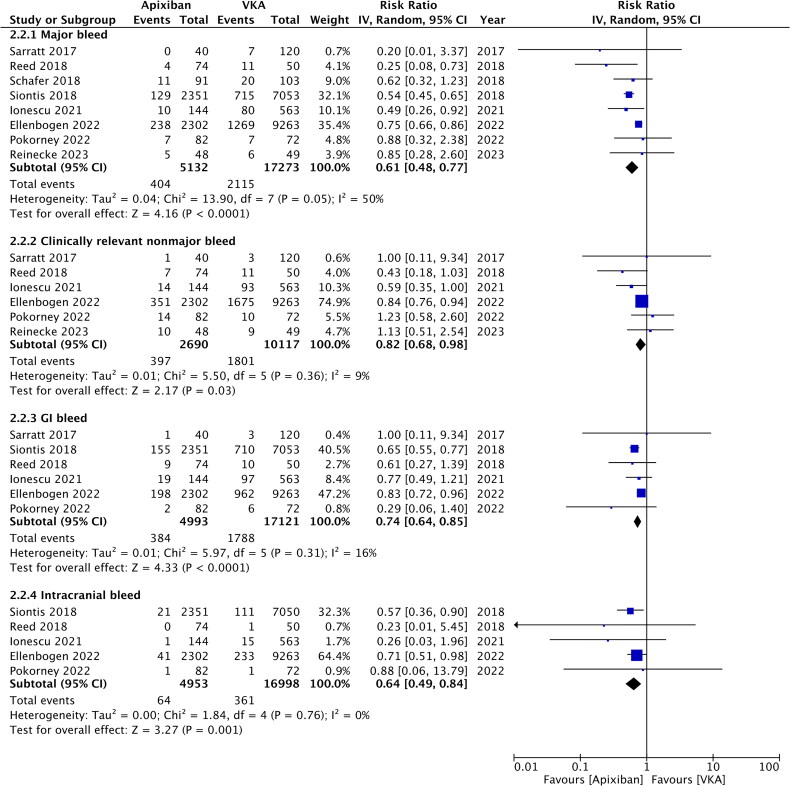
Meta-analysis of bleeding outcomes between apixaban and VKA in patients on dialysis.

The risk of major bleeding was significantly reduced in patients treated with apixaban as compared to VKA (RR: 0.61; 95% CI: 0.48, 0.77; *I*^2^ = 50%) (Figure 2). These results did not change on sensitivity analysis. Likewise, the risk of clinically relevant nonmajor bleeding was found to be significantly lower in the apixaban group (RR: 0.82; 95% CI: 0.68, 0.98; *I*^2^ = 9%) (Figure 2). On sensitivity analysis, exclusion of Sarratt et al. [13] (RR: 0.80; 95% CI: 0.63, 1.03; *I*^2^ = 27%), Ellenbogen et al. [25] (RR: 0.76; 95% CI: 0.51, 1.15; *I*^2^ = 22%), and Reinecke et al. [23] (RR: 0.79; 95% CI: 0.62, 1.00; *I*^2^ = 19%) changed the significance of the results. Meta-analysis also showed that the risk of GI bleeding (RR: 0.74; 95% CI: 0.64, 0.85; *I*^2^ = 16%) and intracranial bleeding (RR: 0.64; 95% CI: 0.49, 0.84; *I*^2^ = 0%) was significantly reduced with apixaban (Figure 2). Both outcomes remained consistent on sensitivity analysis. No major asymmetry was noted on funnel plot (Supplementary Figure 1).

**Figure 3.**
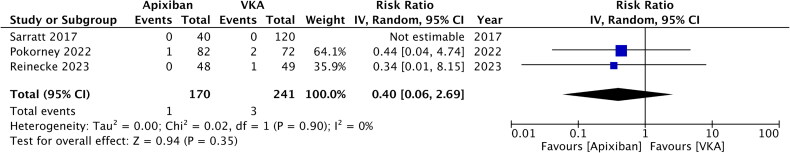
Meta-analysis of ischemic stroke between apixaban and VKA in patients on dialysis.

**Figure 4.**
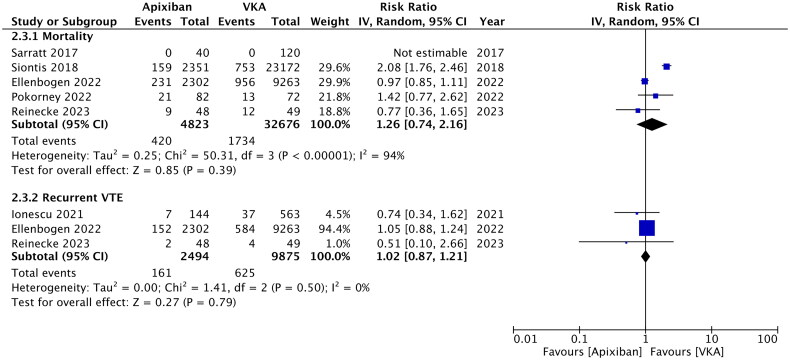
Meta-analysis of mortality and recurrent VTE between apixaban and VKA in patients on dialysis.

Data on ischemic stroke were available from just three studies. Of them, one study did not report any case of stroke. Meta-analysis showed no difference in the risk of ischemic stroke between the two groups (RR: 0.40; 95% CI: 0.06, 2.69; *I*^2^ = 0%) (Figure 3). Similarly, mortality (RR: 1.26; 95% CI: 0.74, 2.16; *I*^2^ = 94%) and risk of recurrent VTE (RR: 1.02; 95% CI: 0.87, 1.21; *I*^2^ = 0%) were comparable in the apixaban and VKA groups (Figure 4). All three outcomes remained unchanged on sensitivity analysis. Publication bias was not detected on funnel plot (Supplementary Figure 2).

### Subgroup analysis

Subgroup analysis for RCTs and non-RCTs is shown in Table 2. Our results show that the risk of major bleeding, clinically relevant nonmajor bleeding, GI bleeding and intracranial bleeding were not significantly different in the apixaban and VKA groups on subgroup analysis of RCTs but remained significant in non-RCTs studies. However, there was no difference in terms of mortality and recurrent VTE between RCTs and non-RCT studies.

**Table 2.** Subgroup analysis based on study design.

Outcome	Study type	Number of studies	Risk ratio [95% confidence intervals]	*I* ^2^
Major bleeding	RCT	2	0.87 [0.41, 1.82]	63
	Non-RCT	6	0.58 [0.45, 0.76]	0
Clinically relevant nonmajor bleeding	RCT	2	1.18 [0.68, 2.05]	0
	Non-RCT	4	0.75 [0.57, 0.97]	22
Gastrointestinal bleeding	RCT	1	0.29 [0.06, 1.40]	–
	Non-RCT	5	0.74 [0.65, 0.84]	13
Intracranial bleeding	RCT	1	0.88 [0.06, 13.79]	–
	Non-RCT	4	0.64 [0.49, 0.84]	0
Mortality	RCT	2	1.09 [0.60, 1.98]	34
	Non-RCT	3	1.42 [0.67, 2.99]	98
Recurrent venous thromboembolism	RCT	1	0.51 [0.10, 2.66]	–
	Non-RCT	2	1.03 [0.87, 1.22]	0

RCT: randomized controlled trial.

### Outcomes of apixaban group based on dosage

Bleeding outcomes, reported by the retrospective studies using different apixaban dosages, are shown in Table 3. A study of Ellenbogen et al. [25] reported hazard ratios of bleeding outcomes in the 2.5 mg group vs. 5 mg group, with no significant difference in bleeding rates. Ionescu et al. [24] reported combined bleeding rates with no difference between apixaban and VKA. In the study of Reed et al. [12], all cases of major bleeding were seen in the 5 mg group while the other studies did not note any difference between the 2.5 mg and 5 mg groups. In two studies, the rates of clinically relevant nonmajor bleed were higher in the 2.5 mg group.

**Table 3.** Outcomes of apixaban group based on drug dose.

Study	Apixaban dose (mg)	Number of patients	Major bleeding	Clinically relevant nonmajor bleed
Ellenbogen et al. [25]	2.5	932	HR: 0.87 (0.65–1.16)	0.98 (0.78–1.25)
	5	1151	Reference	Reference
Ionescu et al. [24]	2.5	92	17 (18.5%)	
	5	52	7 (13.5%)	
Reed et al. [12]	2.5	15	0	4 (26.7%)
	5	59	4 (6.8%)	3 (5.1%)
Siontis et al. [26]	2.5	1317	75 (5.7%)	NR
	5	1034	54 (5.2%)	
Sarratt et al. [13]	2.5	23	0	4 (17.4%)
	5	17	0	1 (5.9%)

HR: hazard ratio; NR: nor reported.

### Risk of bias and certainty of evidence

The results of the risk of bias analysis are shown in Table 4. One RCT had some concerns due to a high loss of follow-up. The other RCTs had a low risk of bias. Most retrospective studies did not adjust for baseline confounding, and therefore, the apixaban and VKA groups were not matched. The NOS score of the studies ranged from 5 to 7. On GRADE assessment, the certainty of evidence was deemed to be very low (Supplementary Table 3).

**Table 4.** Risk of bias analysis.

Study						
Randomized controlled trials	Randomization process	Deviation from intended intervention	Missing outcome data	Measurement of outcomes	Selection of reported result	Overall risk of bias
Reinecke et al. [23]	Low risk	Low risk	Some concerns	Low risk	Low risk	Some concerns
Pokorney et al. [22]	Low risk	Low risk	Low risk	Low risk	Low risk	Low risk

**Table:** 

Observational studies	Selection of cohort	Comparability	Outcome assessment	Newcastle Ottawa Scale score
Ellenbogen et al. [25]	****	**	*	7
Ionescu et al. [24]	****	–	**	6
Schafer et al. [27]	****	–	*	5
Reed et al. [12]	****	–	*	5
Siontis et al. [26]	****	**	**	8
Sarratt et al. [13]	****	–	*	5

Asterisks denote the number of points for each domain (i.e., selection of cohort, comparability and outcome assessment).

## Discussion

The choice of anticoagulation in patients with ESRD and undergoing dialysis has been controversial. Based on the 2014 American Heart Association/American College of Cardiology/Heart Rhythm Society guidelines, warfarin can be used in patients with creatinine clearance of <15 mL/min or in dialysis patients with nonvalvular AF and CHA2DS2-VASc scores ≥2 [29]. However, warfarin can cause vascular calcification, impaired bone mineralization, and reduced bone density which may increase the risk of cardiovascular events and mortality [30,31]. Warfarin also has a very narrow therapeutic window in ESRD patients, with bleeding rates in hemodialysis patients ranging from 0.10 to 0.54 events/person-year [32]. Additionally, patients on dialysis have several risk factors for bleeding such as uremic platelet dysfunction, and comorbid cardiovascular conditions, and are frequently on antiplatelet medications [33]. Therefore, for such patients, there is a great need to develop methods of therapeutic anticoagulation that are effective and are associated with lower risk of major bleeding.

While DOAC is an efficient method of anticoagulation, these drugs have variable renal clearance (e.g., 80% for dabigatran, 33% for rivaroxaban, and 25% for apixaban) [11]. Due to the low dependency on renal excretion, apixaban has become a drug of interest in chronic kidney disease (CKD) and dialysis patients. However, the lack of high-quality evidence has limited the utility of DOAC in such patients and restricted the choice of drugs available to clinicians. Several systematic reviews and meta-analysis studies published in the past few years have analyzed the efficacy and safety of DOAC vs. VKA in patients with renal disease. Xu et al. [34] pooled data from 15 real-world studies to show that DOAC was associated with a small but significant reduction in the risk of stroke or systemic embolism, major bleeding, and mortality when compared to VKA in CKD patients. On subgroup analysis, the decrease in mortality and major bleeding was noted only in moderate-severe CKD cases. Chen et al. [14] combined data from six RCTs and 19 observational studies to examine the difference between DOAC and warfarin in CKD and dialysis patients. As compared to warfarin, DOAC was associated with the 22% reduction in the risk of stroke, systemic thromboembolism, and recurrent VTE, and major bleeding incidence was reduced by 17%. However, efficacy and safety of DOAC were better only in patients with early CKD and became comparable in cases of ESRD or dialysis patients. A major drawback of the review was that only seven studies focused exclusively on dialysis patients. Recently, Mapili et al. [16] conducted a review of just three studies, and noted similar efficacy and safety outcomes of DOAC as compared to VKA in patients with non-valvular AF. Faisaluddin et al. [17] performed a pooled analysis of only RCTs conducted on patients undergoing hemodialysis, and found no difference in the risk of stroke, mortality or major bleeding between patients treated with DOAC and warfarin. Obvious inconsistency in the results of these reviews may be explained by the lack of focus on dialysis patients, combining data of all DOAC, and low number of included studies. In contrast, our study presents high-quality and updated evidence focusing only on apixaban vs. VKA in dialysis patients. Data from two recent RCTs and six retrospective studies were pooled to assess the safety and efficacy of apixaban. We found that apixaban reduced the risk of major bleeding by 39% and clinically relevant nonmajor bleeding by 18% compared to VKA in patients on dialysis. Specifically, the risk of GI and intracranial bleeding was reduced by 26% and 36%, respectively. In terms of efficacy, the data were limited and incoherently reported. Not all studies reported data on stroke, pulmonary embolism, systemic embolism, and recurrent VTE. This underreporting may be due to low sample size and limited follow-ups. The results of our meta-analysis showed that apixaban and VKA had comparable efficacy with no difference in the risk of ischemic stroke, mortality, and recurrent VTE. Importantly, we detected high inter-study heterogeneity regarding major bleeding and mortality. This could be attributed to the variations in the study design, sample size, characteristics of study population, apixaban dose, and follow-up. Due to limited data, we were unable to explore the source of heterogeneity via a meta-regression or multiple subgroup analyses.

An important result noted on subgroup analysis was the difference in outcomes between RCT and non-RCT studies. While studies with the non-RCT design demonstrated that apixaban was associated with the reduction in the risk of all types of bleeding, no such effect was noted in the subgroup of RCTs. Such variation in results points to the effect of selection bias in non-RCTs which may impact study outcomes. It is plausible that clinicians would recommend the newer drug (apixaban) only for patients who have milder disease and less comorbidities. Such patients could therefore present with better outcomes in observational studies. On the contrary, RCT design eliminates such bias and presents highest quality evidence on the efficacy. Therefore, here is a need for further rigorous RCTs to support the current results.

Pharmacokinetic studies have shown that apixaban is well-tolerated in hemodialysis patients. Wang et al. [35] in their study compared a single dose of 5 mg of apixaban in healthy patients with two doses of apixaban 5 mg in hemodialysis patients. The study found that hemodialysis patients had a 36% increase in area under the curve but no increase in *C*_max_ and hemodialysis did not alter apixaban clearance. Another pharmacokinetic steady-state dosing study has shown equivalence in drug exposure with twice daily administration of apixaban 2.5 mg in hemodialysis patients vs. apixaban 5 mg in patients with preserved renal function. However, twice daily administration of apixaban 5 mg in hemodialysis patients increased the therapeutic drug exposure and trough levels [36]. In this review, the studies used a mix of 2.5 mg and 5 mg twice daily dosing of apixaban. Furthermore, most of the studies did not report all outcomes based on the different dosages of the drug. In a qualitative synthesis, we noted that data comparing 2.5 mg vs. 5 mg apixaban in dialysis patients are scarce. Some studies did not report any increased risk of major bleeding with 5 mg dosing while in some studies all cases of major bleeding were confined to the 5 mg group. Also, in two studies, clinically relevant nonmajor bleeds were increased in the 2.5 mg group. Given the limited data, there is a need for further studies comparing the two dosages in dialysis patients.

Our review has certain limitations. Despite being an updated review, the number of studies was not very high. Only two RCTs could be included; the rest were retrospective studies with inherent bias. In retrospective studies, the choice of anticoagulant depends on the preference of the clinician which introduces selection bias. Also, data were primarily reliant on precise entries in medical records. Minor bleeding events may not have been reported leading to inaccurate results. Second, data on international normalized ratio (INR) were not available across most studies. INR represents therapeutic levels of VKA and directly impacts the efficacy and safety of these drugs. Third, there were inconsistent data reporting, especially on stroke, systemic thromboembolism, and recurrent VTE. This led to a low number of studies in our quantitative analysis. Fourth, the dosage of apixaban was not similar across studies and sufficient data were not available for a subgroup analysis. Lastly, there were variations among studies concerning the included population (AF or VTE) and the type of VKA. Most studies used warfarin but one used phenprocoumon. Additionally, the time on dialysis, residual renal function, and urine output were not similar across studies. These are important confounders which can influence outcomes.

The strength of our review is that it is the first to comprehensively present evidence on the efficacy and safety of apixaban vs. VKA in dialysis patients. Compared to previous reviews [7,18] which included just 4–5 studies, we have analyzed data from eight studies to increase the statistical power of the evidence. Patients on dialysis are frequently excluded from RCTs [9,10], and therefore, outcomes of this review provide important evidence on the use of DOAC for such patients. The improved safety and equivalent efficacy of apixaban noted in our meta-analysis widen the anticoagulation armamentarium in patients of dialysis. Apixaban could be routinely used in dialysis patients where the safety of VKA has been questionable.

## Conclusions

Low-quality evidence from a mix of RCTs and retrospective studies shows that apixaban may have better safety and equivalent efficacy as compared to VKA in dialysis patients. The risk of major bleeding and clinically relevant nonmajor bleeding was significantly reduced with apixaban in observational studies but not in RCTs. The predominance of retrospective data warrants caution in the interpretation of results. Further RCTs are needed to improve the quality of evidence.

## Supplementary Material

Supplemental Material

## Data Availability

The datasets used and/or analyzed during the current study are available from the corresponding author on reasonable request.
