# Supplementary figures and images for: Safety and efficacy of apixaban versus vitamin K antagonists in patients undergoing dialysis: a systematic review and meta-analysis
Source: Ren Fail. 2024 May 21;46(1):2349114. doi: 10.1080/0886022X.2024.2349114 (PMC11110875; doi:10.1080/0886022X.2024.2349114)

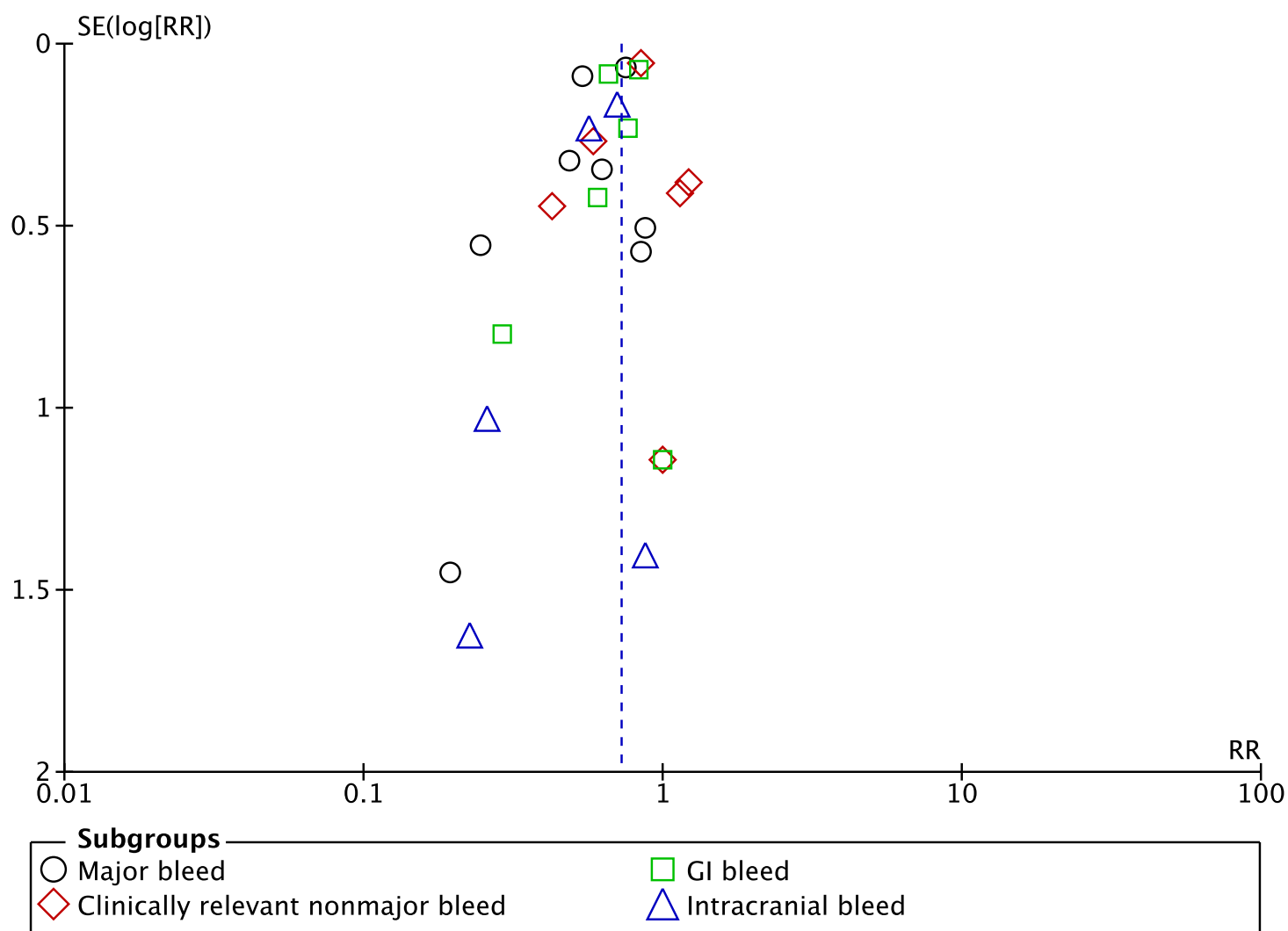

Supplement: Supplemental Material [file IRNF_A_2349114_SM9058.zip › Supplementary Figure 1.pdf]

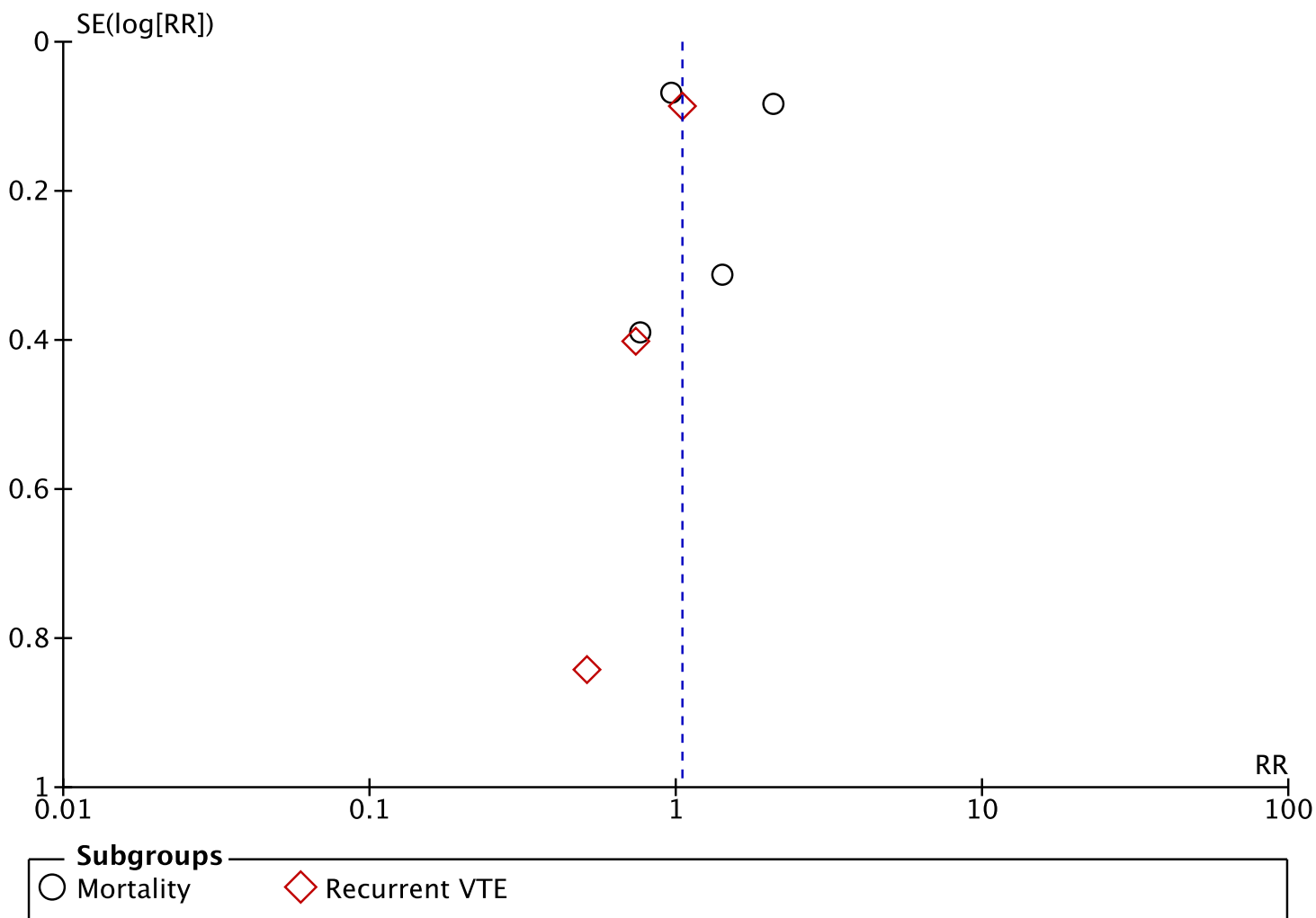

Supplement: Supplemental Material [file IRNF_A_2349114_SM9058.zip › Supplementary figure 2.pdf]
